# Virtual Reality for Motor and Cognitive Rehabilitation From Clinic to Home: A Pilot Feasibility and Efficacy Study for Persons With Chronic Stroke

**DOI:** 10.3389/fneur.2021.601131

**Published:** 2021-04-07

**Authors:** Johanna Jonsdottir, Francesca Baglio, Patrizia Gindri, Sara Isernia, Carlotta Castiglioni, Cristina Gramigna, Giovanna Palumbo, Chiara Pagliari, Sonia Di Tella, Gloria Perini, Thomas Bowman, Marco Salza, Franco Molteni

**Affiliations:** ^1^IRCCS Fondazione Don Carlo Gnocchi ONLUS, Milan, Italy; ^2^Ospedale San Camillo, Turin, Italy; ^3^Ospedale Valduce, Villa Beretta, Costa Masnaga, Italy

**Keywords:** stroke, hemiplegia after stroke, virtual reality, rehabilitation, continuity of care, mobility, cognition

## Abstract

**Aims:** Continuity of care is an important issue in healthcare for persons after stroke. The present multi-center pilot study investigates the feasibility and efficiency of an innovative approach, the Human Empowerment Aging and Disability (HEAD), for digital-health motor and cognitive rehabilitation. The approach is explored within an in-clinic context (ClinicHEAD) and in continuity of care (HomeHEAD) for persons after chronic stroke.

**Methods:** Thirty-four outpatients with chronic stroke (mean age 55 years, SD 13.7) participated. The HEAD VR protocol was administered in two consecutive phases: Phase I in clinic (ClinicHEAD) consisting of 4 weeks of 12 supervised HEAD rehabilitation sessions (45-min), including motor, cognitive and dual task for all participants; Phase II at home (HomeHEAD) consisted of 60 sessions of the same VR activities, 5 times/week for 3 months. All participants in the ClinicHEAD were allocated (ratio 1:2) to continue with tele-monitored home rehabilitation (HH, *N* = 11) or to follow usual care (UC, *N* = 23). Blind evaluation was carried out at baseline, after ClinicHEAD, after 3 months of HomeHEAD and at 3 months Follow-up. Primary outcomes were functional mobility [2-min Walking Test (2MWT)] and cognition [Montreal Cognitive Assessment (MoCA)]. Feasibility and acceptance were assessed with adherence to treatment and the System Usability Satisfaction. Within group analyses were done with dependent samples *t*-tests, and between groups HomeHEAD comparisons were carried out on change scores with independent samples *t*-test (*p* = 0.05, two tailed).

**Results:** The HEAD protocol was feasible with good adherence both in the ClinicHEAD phase (92%) and HomeHEAD (89%) phase, along with good perceived system satisfaction. ClinicHEAD resulted in a significant increase in functional mobility (2MWT, *p* = 0.02) and cognition (MoCA, *p* = 0.003) and most secondary outcome variables. At 3 months follow up of HomeHEAD the HH_group showed a further significantly greater maintenance of functional mobility with respect to UC_group (*p* = 0.04).

**Conclusion:** The HEAD VR protocol was feasible in clinical and at home tele-rehabilitation for persons in the chronic phase after stroke. In clinic the approach was effective in augmenting motor and cognitive abilities and at home it was effective in longterm maintenance of functional mobility, indicating its usefulness in continuity of care.

**Clinical Trial Registration:** ClinicalTrials.gov, NCT03025126.

## Introduction

Neurological disorders, including post-stroke sequelae, are among the most common causes of longterm disability in the general population. Persons with hemiplegia after stroke are faced with multifactorial motor and cognitive disabilities making longterm neurorehabilitation crucial to prevent disease aggravations and enhance their activity levels and quality of life (QoL) (1, 2). Most moderate to serious stroke sequelae require periodic sessions of rehabilitation, or even hospitalization, making maintenance of results an essential aspect (2). Nonetheless, not all persons in the more chronic phase post-stroke can have access to longterm continuous rehabilitation, leading to non-optimal recovery and reduced functionality that further impacts upon participation in life situations.

The integration of Digital Health (DH) approaches, including innovative exercises performed in a virtual reality environment within a home rehabilitation program, are an attractive solution to continuity of care and can constitute a functional low-cost resource for monitoring and applying rehabilitation in new motivating ways (3). Virtual reality training has been implemented for balance training, for improving arm function and for cognitive training in persons with stroke (4–7).

Systematic reviews have demonstrated that rehabilitation incorporated in VR technology is feasible and sometimes even more effective than standard rehabilitation for improving motor and cognitive symptoms after stroke and that they can result in potentially better community integration (8–11). However, motivation and adherence to home rehabilitation protocols remain a concern making the setup of interesting DH approaches essential for the success of the approach (12–15). The inclusion of the gaming concept in rehabilitation has been demonstrated to make the clinical program more motivating and immersive, an important concept in continuation of rehabilitation care (16–19). Also, short video clips have historically been used to elicit emotion and motivate people with interesting results, indicating that their dynamic nature may provide a model more representative of reality (20, 21). Video clips that are meaningful to the person and incorporated in a rehabilitation gaming concept may be particularly motivating and useful for addressing the various motor and cognitive stroke sequelae that persons face post-stroke and during lifetime degenerative neurological disorders.

The Human Empowerment Aging and Disability program (HEAD), a virtual reality Digital Health neurorehabilitation to maintain and improve motor and cognitive function in persons with neurological disorders, was developed combining these two motivating approaches (22). The HEAD approach is thus based on the use of low-cost devices and multimedia content, including short motivating video clips of Radiotelevisone Italiana (RAI) programs within the context of VR serious gaming neurorehabilitation. The specific purpose of the present pilot study was to provide the initial evidence of the longterm effect of this innovative way of applying motor and cognitive rehabilitation administered first in a supervised way and then individually at home. The HEAD VR neurorehabilitation was applied in a multicenter study, the first 4 weeks under supervision in the clinic as ambulatory services (ClinicHEAD) and immediately after at home for 3 months (HomeHEAD). Participants in the study were persons with Parkinson's disorders (PD), Multiple sclerosis (MS) and persons in the chronic phase post-stroke. With this study setup the end users, therapists and patients/clients, learned how to use the system and problemsolve so that once in the home they were already familiar with the HEAD system. Feasibility aspects of the intervention for persons with PD, MS and post-stroke have already been published in Isernia et al. (22) and were found to be good, with high adherence and good perceived functioning in routine and participation in daily life, and a generally satisfying feedback regarding the acceptance of the HEAD technology. Further, recently published (23) results for persons with PD using the intervention were promising, in that the HEAD program resulted in improved motor and cognitive abilities after the ClinicHEAD and in preserved motor and non-motor function at follow up.

The present study focuses on outpatients with chronic stroke sequelae that participated in the HEAD approach. The improvement in various motor and cognitive functions, during a HEAD telerehabilitation carried out in the clinic and supervised by therapists and psychologists, will be verified for all participants with stroke. Consequently, difference in outcomes between those that continue with the HEAD rehabilitation care at home and those that follow usual care will be explored. Both at the end of the HomeHEAD rehabilitation period at 3 months after ClinicHEAD, and at follow up 6 months after ClinicHEAD.

The main hypothesis is that following a supervised outpatient HEAD telerehabilitation people with stroke that continue with the HEAD telerehabilitation approach at home for 3 months will maintain the effects better at the end of the follow up than those that proceed with usual care.

## Methods

### Participants

The study was carried out as a multicenter study and included three different neurological disorders, however, in the present study only data on participants that were outpatients post-stroke will be reported upon. Forty five persons, outpatients post-stroke, were consecutively recruited from 3 Italian clinical Centers: the Rehabilitation Center Villa Beretta of Lecco, the IRCCS Don Carlo Gnocchi Foundation of Milan, and District Clinic San Camillo of Turin. The study period lasted from March 2016 to December 2017.

The study protocol was approved by the local Ethical Committees of the three centers involved (Comitato Etico IRCCS Fondazione Don Gnocchi, Comitato Etico of the inter-company of Lecco, Como and Sondrio, Comitato Etico of the inter-company “Città della Salute e della Scienza” of Turin) and all subjects provided written and informed consent prior to participation in the study.

Inclusion criteria for the persons post-stroke was the following: age range of 18–80 and stroke in the chronic phase, at least 6 months after the acute event. Exclusion criteria included: (a) Mini Mental State Examination (MMSE) (24) score <20; (b) the presence of disabling pain; (c) upper limb limited passive range of motion; (d) epilepsy; (e) severe deficit of visual acuity and auditory perception; (f) presence of severe deficit in communication and severe dysmetry.

The data analyzed and presented here is from a subgroup of the study participants that met the criteria of 80% adhesion to the study protocol and that could stand, even with support, for 30 s. The first criteria were used to respect the minimum rate of adherence needed to appraise the quality of the clinical trial (25) and the second because the primary motor outcome was the 2-minute walking test. This resulted in two persons being excluded from the analysis.

### Study Design

The study was carried out in two steps: ClinicHEAD (Phase I) and HomeHEAD (Phase II). The ClinicHEAD consisted of a pre-post study to test the intervention delivery characteristics (safety, feasibility and acceptability, and appropriateness of measurements) and was carried out in the clinic [see Isernia et al. (22) for more detailed information]. Persons with stroke sequelae were consecutively recruited from persons that were requesting outpatient rehabilitative services from the respective centers. After enrollment and baseline assessment, they were all assigned into the ClinicHEAD program (Phase I) and received 12 sessions of VR training over a period of 4 weeks in-clinic outpatient services. During the month of in-clinic treatment the HEAD sessions (45 min three times per week) the participants were supervised by health professionals. Activities, repetitions and level of difficulty were tailored to each participant's abilities and constantly updated. Participants were encouraged to access the HEAD platform independently in order to develop problem solving techniques. The second part of the study was carried out as telerehabilitation in the home of the participants (HomeHEAD, Phase II) and consisted in a single-blind (observer), interventional, two-treatment arms (HomeHEAD vs. Usual-Care) controlled clinical trial. At the end of the ClinicHEAD the participants were consecutively assigned into either a HomeHEAD group [12 weeks of HEAD telerehabilitation (HH) at home] or a usual care group [12 weeks of Treatment as Usual (Usual Care, UC) at home] by a person outside of the study with a ratio of 1:2. This ratio was based on the pilot nature of the study and a limited number of available HomeHEAD technological kits for the time period of the study. This Phase II of the study served for an initial verification of efficacy of the approach compared to usual care in maintenance of in-clinic results and to evaluate the occurrence of adverse events.

Further details of the study protocol are given elsewhere [see Isernia et al. (22)].

#### Outcome Measures

Outcome measures were collected by researchers blinded to group allocation (observer-blind). Since participants could not be blinded to their treatment allocation, they were instructed not to discuss the nature of their intervention with the health professionals doing the assessments. Primary and secondary outcome measures were obtained: (a) at baseline (T0) before starting the 12 ClinicHEAD sessions; (b) at the end of the ClinicHEAD (T1) before starting the in-home HEAD sessions or the Usual care; (c) at post-HomeHEAD or Usual care, 3 months after (T2); (d) at follow up 7 months after baseline (T3).

**Figure 1:**
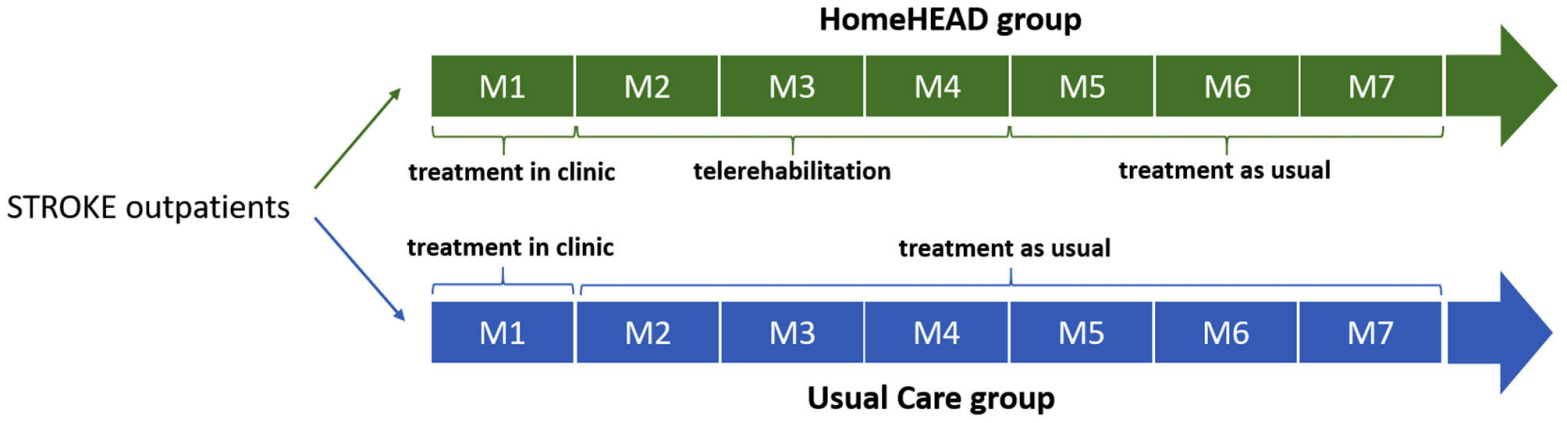
Timeline of the HEAD trial in months.

The study timeline is illustrated in Figure 1.

This was a feasibility and pilot interventional trial used to identify the appropriateness of the outcome measures, and to generate effect sizes for a Phase III trial. The data analyzed here is of 34 persons that received the VR treatment during ClinicHEAD and HomeHEAD, and met the adhesion and standing criteria.

### Intervention ClinicHEAD and HomeHEAD

The ClinicHEAD training was supervised by physical therapists and psychologists. The training was carried out with Kinect (Microsoft, WA, USA) and Leap Motion (Leap Motion Inc., CA, USA) in a room with an area of 20 m^2^, three times a week for 4 weeks. The image was projected on a television screen with the participants placed in front with ample space to carry out the exercises. Instrumentation is described more in detail in Isernia et al. (22).

Motor, cognitive and occupational exercises were integrated in a paradigm of VR activities. Activities were coarsely divided into those that were more of type motor activities, cognitive activities or occupational activities. Motor activities included unilateral and bilateral arm movements, equilibrium exercises involving trunk movements, unilateral stance, reduced base of support and in place gait activities and leg movements. Cognitive activities requiring attention, memory, executive function and so on required hand movements for responses and also occupational activities, such as shaving, putting on make-up, doing a puzzle etc.

Each activity started with a short movie that was then interrupted periodically with motivating breaks that called for rehabilitative activities. Specifically, in the motor activities the movie was stopped in different moments of the movie clip. At each movie break a serious game, implying a rehabilitation activity, took place. The number of intervals were from 2 to 7 for each movie clip and were related to the difficulty level of the planned activities. Moreover, within the single movie break, the virtual activity requested a variable number of repetitions for the specific neuromotor exercise set according to the intensity level of the rehabilitative activities programmed. During cognitive activities attention had to be payed to the content of the movie, requiring, for example, information to be memorized. The movie clips came from the historical collection of RAI documentaries and movies, and the movie clips shown were tailored to the participant's particular interests.

#### HomeHEAD Phase II

The HomeHEAD part followed the same principles of the ClinicHEAD but was carried out in the home of the participant without supervision. Training was programmed to be carried out five times per week for ~45 min, and once per week the trained physical therapists and psychologists modified the program for the following week according to participants abilities. The HomeHEAD differed from the ClinicHEAD in that all motor and cognitive activities were carried out in a sitting position. The participants were invited to call the health personnel in case of difficulties with the setup or questions regarding the carrying out of exercises.

The UC participants were asked to not participate in physical activities different from those that they would usually do during the protocol duration.

Both the active group participants and the usual care participants were invited to follow health recommendations of their physician or neurologist for their clinical conditions.

### Assessment Design and Outcome Measures

The assessment protocol consisted in multi-domains evaluation of feasibility, physical activity, motor and cognitive abilities, and QoL.

Feasibility of the intervention was assessed by participant adherence and perceived ease of use of the HEAD system. For perceived ease of use The System Usability Scale (SUS) (26) was administered at T1 (to all particpants) and T2 (only to HomeHEAD users). The scale consists of a 10-item scale with a score of 100 representing a perfect facility of use. A cut-off score indicating a satisfying level of usability is 68. Adherence was assessed as the percentage of planned sessions actually performed. Mean duration of motor and non-motor activities performed per session are also reported. Evaluation of motor and cognitive functions was carried out at T0, T1, T2, and T3 and was comprised of the following motor abilities and neuropsychological tests.

Two Minute Walk Test (2MWT) (27) and 10 Meter Walk Test (10MWT) (28) for evaluation of gait resistance and gait speed; Berg Balance Scale (BBS) (29), a test for the assessment of patient's static balance, and Box and Blocks Test (BBT) (30) for evaluating participant's dexterity and arm function; Motricity Index (MI) (31), an index for assessing the strength of key muscle groups in upper and lower limbs post-stroke. The Montreal Cognitive Assessment (MoCA) (32), a sensitive tool for global cognitive level assessment; Rivermead Behavioral Memory Test-Third Edition (RBMT-3) (33), an ecological battery for assessment of memory abilities.

Primary outcomes were the 2MWT and the MoCA. All other variables were treated as secondary outcomes.

### Data Analysis

Statistical analysis was performed using Statistica software. Descriptive statistics were employed to evaluate efficiency and effectiveness. Normal distribution of variables was checked through the Kolmogorov-Smirnov normality test.

Intention-to-treat-analyses were used for all outcomes. If an individual's evaluation was missing at any assessment point, the individual's outcome of the last evaluation done was carried forward.

Efficacy of the HEAD intervention in the ClinicHEAD Phase within the whole group was verified with *t*-tests. Specifically, paired sample *t*-tests were performed to compare T1 vs. T0 outcome measures in the whole sample, and T2 vs. T1 in the HomeHEAD group and the UC group. For group comparisons we computed the change at T2 and T3 relative to beginning of HomeHEAD (T1) through calculation of change scores (Δvalues) from T2-T1 to T3-T1, and after that we adopted independent sample *t*-tests comparing HomeHEAD and UC groups' change values at 3 months and 6 months follow up of HomeHEAD.

Effect size of within group differences were calculated for ClinicHEAD. Effect sizes were interpreted as trivial (*d* < 0.2), small (0.2 *d* < 0.5), moderate (0.5 *d* < 0.8), and large (*d* > 0.8) (34, 35).

Results were considered statistically significant when *p*-value was < 0.05, tests were two-tailed.

Estimation of sample sizes for a future study with adequate power was carried out on 2MWT results from T0 and T3 evaluations.

## Results

Thirty-four participants meeting the adhesion and standing criteria finished the Phase I ClinicHEAD (mean age 59 years (SD 13.6). All 34 persons that finished ClinicHEAD finished also the 3-month Phase II assessment (HomeHEAD *N* = 11 and Usual care *N* = 23 (see Table 1). Five persons in the UC_group were not available for follow up assessments at 6 month after ClinicHEAD due to difficulties in arriving to the Institutes for evaluation. Their data was treated as intention-to-treat for all analyses.

**Table 1:** Demographics and baseline characteristics of the ClinicHEAD and the HomeHEAD groups (UC and HH).

	**ClinicHEAD**	**UC**	**HH**	**UC vs. HH**
				***P***
*N*	34	23	11	
	Mean (SD)	Mean (SD)	Mean (SD)	
Age (Years)	59.00 (12.68)	60.19 (9.63)	56.72 (17.4)	0.365
Education [Mean (SD)]	13.56 (3.49)	12.71(3.16)	15.18 (3.65)	**0.028**
Sex (M/F)	18/14	13/8	5/6	0.465
Affected arm right/left	14/18	8/13	6/5	0.465
**Motor functioning**				
2MWT (Meters)	75.48 (45.83)	76.06 (52.8)	74.27 (35.26)	0.917
BBS	40.15 (15.58)	39.39 (16.14)	41.73 (14.95)	0.689
10MT (seconds)	15.67 (12.52)	17.38 (14.52)	12.09 (5.7)	0.255
MI_A	61.60 (26.35)	58.06 (26.35)	69 (25.34)	0.264
MI_NA	99.88 (0.68)	99.83 (0.83)	100.0 (0)	0.498
BBT—affected	15.4 (19.45)	11.87 (16.39)	21.82 (23.08)	0.157
BBT—non-affected	47.81 (12.31)	45.35 (8.57)	53.36 (16.17)	0.066
**Cognitive functioning**				
MoCA	22.56 (4.66)	21.91 (4.16)	23 (5.8)	0.536
RBMT-GMI	80.9 (17.42)	79.91 (17.46)	80.64 (17.7)	0.911

*p-values < 0.05 are reported in bold; UC, usual care; HH, HomeHEAD; t2MWT, 2 m Walking Test; BBS, Berg Balance Scale; 10MT, 10 m Walking test; MI_A, Motricity Index Affected side; MI_NA, Motricity Index Not-Affected side; BBT, Box and Block Test; MoCA, Montreal Cognitive Assessment; RBMT-GMI, Rivermead Behavioral Memory Test-Third Edition—Global Memory Index; M, Mean; SD, Standard Deviation*.

No study related adverse events were reported in the HH_group.

There were no statistically significant differences between the HH_group and UC group regarding age and onset while educational level resulted different. See Table 1 for demographics and characteristics of the whole sample and the two subgroups.

### Feasibility

Adherence to the supervised ClinicHEAD was 92% of planned rehabilitation sessions while to the HomeHEAD it was 89%.

Out of 60 sessions maximum programmed for the HomeHEAD, an average of 55 (SD13.7) sessions were carried out. Mean daily time spent in VR activity was 35.3 min (SD 6.25), of which 18.6 (4.6) min were spent in motor activities and 10.7 min (3.7) and 6.3 min (3.4) were spent, respectively, in more cognitive and occupational activities. Over time of using the system there was an increase in perceived satisfaction with the system as measured by the SUS, after the intervention period the median total score of the 11 participants that continued to use the system at home (HomeHEAD, HH_group) was 77.5/100 (IQR 67.5–82.5) with learnability and usability subscores of 3.0 (IQR 2.5–4) and 3.0 (IQR 2.6–3.5) respectively. For more detailed information on system satisfaction for all neurological patients see Isernia et al. (22).

### Efficiency

#### Changes in Outcome Measures After ClinicHEAD (T1-T0, *N* = 34)

Following the supervised ClinicHEAD sessions executed by the whole group, there were statistically significant improvements in both the primary motor outcome (2MWT: *t* = 2.684; *df* = 33; *p* = 0.011; Cohen's *d* = 0.894) and in the primary cognitive outcome (MoCA: *t* = 3.644; *df* = 33; *p* = 0.001; Cohen's *d* = 1.253). See Table 2 for outcome values of ClinicHEAD.

**Table 2:** Efficiency of the ClinicHEAD approach (T1-T0).

	**T0 *N* = 34**	**T1 *N* = 34**		
	**Mean (SD)**	**Mean (SD)**	***P***	**Cohen's *D***
**Primary outcome**				
2MWT	75.48 (45.83)	82.95 (47.68)	**0.011**	0.894
MoCA	22.26 (4.69)	23.94 (4.2)	**0.001**	1.253
**Secondary outcome**				
BBS	40.15 (15.58)	41.78 (15.46)	**0.010**	1.067
10MWT	15.67 (12.52)	14.46 (12.17)	**0.005**	1.071
MI_A	61.60 (26.35)	64.65 (25.19)	**0.044**	0.595
MI_NA	99.88 (0.68)	100 (0)	0.324	0.349
BBT—affected	15.09 (19.05)	15.85 (20.30)	0.265	0.298
BBT—non-affected	47.81 (12.31)	49.21 (13.31)	**0.033**	0.639
RBMT-GMI	80.9 (17.42)	84.72 (19.2)	**0.032**	0.817

*p-values < 0.05 are reported in bold. UC, usual care; HH, HomeHEAD; 2MWT, 2-m Walk Test; MoCA, Montreal Cognitive Assessment; BBS, Berg Balance Scale; 10MWT, 10 m walking test; MI_A, Motricity Index Affected side; MI_NA, Motricity Index Not-Affected side; BBT, Box and Block Test; RBMT-GMI, Rivermead Behavioral Memory Test-Third Edition—Global Memory Index; SD, Standard Deviation*.

Regarding secondary motor outcomes, there were statistically significant improvements in balance (BBS: *t* = 2.722; *df* = 33; *p* = 0.010; Cohen's *d* = 1.067); in gait velocity (10MWT: *t* = −2.962; *df* = 33; *p* = 0.006; Cohen's *d* = 1.071); memory (RBMT: *t* = −2.253; *df* = 33; *p* = 0.031; Cohen's *d* = 0.817); motor function of the affected side (MI: *t* = 2.094; *df* = 33; *p* = 0.043; Cohen's *d* = to do) and in ability of the non-affected arm (BBT: *t* = 2.227; *df* = 33; *p* = 0032; Cohen's *d* = 0.639).

#### HomeHEAD (T1-T2-T3)

At discharge the ClinicHEAD group was randomized 1:2 into the groups HH (*N* = 11) and UC (*N* = 21) with baseline characteristics of the two treatment groups being similar in terms of age and onset of stroke (*P* > 0.05) while in terms of educational level they differed by 0.85 years of education (*p* = 0.03). Regarding differences at baseline of the HomeHEAD phase all group specific outcome values at all time points are given in Table 3. The two groups were balanced in motor, cognitive and qualitative measures (*P* > 0.05) at T1 while there was a statistically significant difference on the Box and Block Test of the non-affected arm (*p* = 0.011).

**Table 3:** Means and SD of the HomeHEAD outcomes (T1, T2, and T3).

	**T1**		**T2**		**T3**	
	**HH**	**UC**	**HH**	**UC**	**HH**	**UC**
	**Mean (SD)**	**Mean (SD)**	**Mean (SD)**	**Mean (SD)**	**Mean (SD)**	**Mean (SD)**
**Primary outcome**						
2MWT	84.36 (33.57)	82.28 (53.82)	92.45 (40.4)	80.69 (53.19)	90.63 (44.1)	76.35^*^ (47.85)
MoCA	23.45 (4.34)	24.17 (4.21)	24.36 (4.24)	23.13 (4.21)	23.27 (5.92)	23.55 (4.22)
**Secondary outcome**						
BBS	43.37 (15.07)	41.65 (15.47)	42.27 (15.84)	40.43 (15.87)	43.45 (14.55)	39.26 (16.78)
10MWT	10.3 (6.23)	16.45 (13.85)	10.41 (7.44)	17.74 (15.2)	10.82 (7.03)	18.24 (15.38)
MI_A	72.04 (18.23)	61.11 (27.57)	70.63 (22.45)	63.12 (27.57)	76.27 (23.52)	61.74 (27.41)
MI_NA	100.0 (0)	100.0 (0)	99.64 (1.21)	99.83 (0.83)	100.0 (0)	100.0 (0)
BBT-affected	22.18 (23.62)	12.83 (18.31)	22.9 (24.26)	13.65 (20.14)	24.27 (25.36)	13.78 (20.12)
BBT-non affected	54.18 (16.77)	47.30^§^ (10.46)	53.9 (18.16)	48.56 (9.67)	57.45 (18.58)	49.83 (10.26)
RBMT-GMI	88.27 (18.75)	82.56 (18.76)	91.64 (18.62)	86.13 (20.73)	93.27^**^ (19.17)	88.6 (20.18)

* *p < 0.05, significant worsening T1-T3*;

** *p < 0.05, significant improvement T1-T3*;

§ *p < 0.05, significant differences between groups at T1; UC, usual care; 2MWT, 2-min Walk Test; MoCA, Montreal Cognitive Assessment; BBS, Berg Balance Scale; BBT, Box and Block Test; 10MWT, 10 m walking test; MI_A, Motricity Index Affected side; MI_NA, Motricity Index Not-Affected side; RBMT-GMI, Rivermead Behavioral Memory Test-Third Edition—Global Memory Index; SD, Standard Deviation*.

##### Motor and Cognitive Outcomes

*Within Group Differences (T2-T1) and Comparison Between HH and UC Group at End of HomeHEAD Intervention (*Δ*T2-T1)*. See Table 3 for outcomes for T1 to T2 and T3, and Table 4 for change scores and statistical significance.

**Table 4:** Comparison between UC and HH groups on neuropsychological and motor measures after 3-months of HomeHEAD/UC (ΔT2-T1) and after 6-months from ClinicHEAD (ΔT3-T1).

	**ΔT2-T1**			**ΔT3-T1**		
	**HH**	**UC**		**HH**	**UC**	
	**Mean (SD)**	**Mean (SD)**	***p***	**Mean (SD)**	**Mean (SD)**	***P***
**Primary outcome**						
2MWT	8.09 (20.19)	−1.59 (9.73)	0.066	6.27 (20.19)	−5.93 (11.63)	**0.032**
MoCA	−1.09 (2.34)	0.48 (2.1)	0.059	−0.18 (2.68)	−0.61 (2.82)	0.678
**Secondary outcome**						
BBS	−1.09 (5.17)	−1.22 (5.56)	0.95	0.09 (9.33)	−2.39 (8.44)	0.443
10MWT	0.10 (1.88)	1.29 (4.69)	0.899	0.51 (2.89)	1.79 (4.94)	0.434
MI_A	−1.41 (10.0)	2.01 (11.9)	0.41	5.64 (7.51)	−1.38 (9.09)	**0.034**
MI_NA	−0.36 (1.21)	−0.17 (0.83)	0.59	0.36 (1.21)	0.17 (0.83)	0.596
BBT_A	0.73 (1.74)	0.82 (2.60)	0.91	2.09 (3.7)	0.95 (2.8)	0.324
BBT_NA	−0.27 (5.68)	1.26 (5.78)	0.91	3.27 (6.02)	2.52 (5.39)	0.380
RBMT-GMI	3.36 (10.93)	3.56 (14.85)	0.968	5.00 (3.74)	6.04 (16.11)	0.834

*p-values < 0.05 are reported in bold. HH, HomeHead; UC, usual care; HH, HomeHEAD; 2MWT, 2-min Walk Test; MoCA, Montreal Cognitive Assessment; BBS, Berg Balance Scale; 10MWT, 10 m Walking Test; MI_A, Motricity Index Affected side; MI_NA, Motricity Index Not-Affected side; BBT, Box and Block Test; RBMT-GMI, Rivermead Behavioral Memory Test-Third Edition—Global Memory Index; SD, Standard Deviation*.

Regarding the efficacy of the HomeHEAD, after 3 months of the VR approach the HH_group maintained the benefit from ClinicHEAD with no significant improvement nor decline in any outcome variable (T2-T1; *p* > 050). Similarly, in the UC_group there was maintenance of the positive effects of the ClinicHEAD with no significant change in any outcome variable (T2-T1; *p* ≥ 0.05).

Between groups analysis through change scores revealed no significant difference in change scores of primary or secondary outcomes between the HH_group and UC_group from the beginning of HomeHEAD (T1) to end of the 3 months of HomeHEAD (T2) (ΔT2-T1; *p* > 0.05).

##### Within Group Differences (T3-T1) and Comparison Between HH and UC Group at HomeHEAD Follow Up (ΔT3-T1)

See Table 2 for outcomes for T1 to T2 and T3, and Table 3 for change scores and statistical significance. Regarding the maintenance of the HomeHEAD effect, at the 3 months follow up after HomeHEAD the HH_group had a significant improvement in memory (RBMT: T3-T1 *t* = 3.741; *df* = 10; *p* = 0.001) with respect to T1 and overall maintained the benefit from ClinicHEAD with no significant improvement nor decline in any other outcome variable. At the 3 months follow up after HomeHEAD the UC_group had a significant decline in functional mobility (2MWT: *T3-T1; t* = −2.446; *df* = 22; *p* = 0.02) with respect to T1 while overall there was maintenance of the positive effects of the ClinicHEAD with no significant change in any other outcome variable (*p* ≥ 0.05).

Between groups analysis through change scores revealed significant difference in change scores of primary outcome functional mobility between the HH_group and UC_group from the beginning of HomeHEAD (T1) to end of follow up period (T3) (2MWT: ΔT3-T1: *t* = −2.242; *df* = 32; *p* = 0.032) with the HH_group showing improvement and the UC group detoriation of the parameter; and similarly, in secondary outcome motricity of the affected site (MI: ΔT3-T1: *t* = −2.21716; *df* = 32; *p* = 0.034).

Sample size estimation for a future study, assuming a value for alpha of 0.05 and a desired power of 0.8, with mean increases on the 2MWT of 0.28 meters for the UC_group and 13.36 meters for the HH_group (T3-T0) and pooled standard deviation, revealed the need for at least 38 persons with chronic stroke per group.

## Discussion

The present multi-center pilot study investigated the feasibility and preliminary efficacy of an innovative VR approach including short motivating video clips from RAI programs within the context of neurorehabilitation in clinic (ClinicHEAD) and in continuity of care (HomeHEAD). The approach was feasible and the technical complexity was acceptable to the participants that were in the chronic phase after stroke. Following the first supervised ClinicHEAD phase executed by all participants there was an overall improvement in most motor and cognitive domains. At the end of the 3 months HomeHEAD phase there was good compliance to the HomeHEAD protocol and both groups preserved their ClinicHEAD results. However, there was a better preservation of mobility in the HH_group at the end of the 3 months HomeHEAD follow up period.

Participants were on the average 15 months post-stroke and were quite heterogeneous in their functional and cognitive abilities. Adherence was good in both the ClinicHEAD and the HomeHEAD phase with satisfying feedback regarding system usability and learnability. The adherence of our stroke participants was in line with that reported in the literature (36). Importantly, there were no adverse events registered in either rehabilitation phase and all points to the HEAD VR approach being a safe, doable and motivating approach to neurorehabilitation in continuity of care. The inclusion of the video clips and the weekly adjustment of exercises according to abilities in the HEAD approach may have positively influenced the adherence.

### ClinicHEAD (T1-T0)

Following the 12 sessions of ClinicHEAD we saw improvements in most aspects of motor and cognitive abilities. Although there was great heterogeneity in walking abilities of the participants, after the first 12 supervised sessions there was an overall improvement in gait velocity and gait resistance of about 20%, indicating that the ClinicHEAD approach was beneficial for walking activities. Even if there was no direct overground walking or training the HEAD protocol included dynamic activities in standing, such as, walking on the spot and knee raises relative to virtual activities of walking and stair climbing, that appear to have been beneficial for overground walking abilities of the participants. The improvement seen in real life mobility after a month of the HEAD VR protocol is promising and is in line with the results of several literature review on the effect of VR protocols on gait and balance in persons post-stroke (4, 6, 9, 12). The results also concord with recent literature reporting on trials using a VR approach to rehabilitation of gait and balance (37, 38).

Although cognitive impairment is common in the chronic phase after stroke and there is an evident connection between cognitive and motor deficits the impact of combining motor and cognitive aspects in VR approaches has been poorly investigated (3, 39). Following ClinicHEAD we saw small but significant positive changes (<10%), both in global cognitive functions and memory. These cognitive benefits are in line with findings from a random controlled VR trial carried out by Faria et al. (40) and a couple of reviews looking at both motor and cognitive outcomes after VR approaches (41, 42). Both reviews found a small to medium effect favoring a VR approach compared to conventional therapy with bigger effects on motor outcomes similar to that seen in our study. The above gives support to combining motor and cognitive training in VR approaches.

The HEAD approach did, however, not impact on hand function. This may be because there was great heterogeneity in affected arm abilities of our study participants and it should be noted that the participants that were more severely affected often used the not affected limb during the VR rehabilitation resulting in little or no training of the affected arm and hand. Further, even when the affected arm was used for the VR activities, the amount of time spent in arm activities was only about 50%, the rest of the activities were focused on trunk and lower limb activities. This may have influenced the interventions efficacy on arm function since intensity and repetition are especially important in arm rehabilitation for persons with chronic stroke (43). Our results add to controversial results of other studies on VR and game applications that have been used to rehabilitate arm function in persons with chronic stroke (9, 11).

The present study was a pilot study and the ClinicHEAD was carried out on all participants with no control group, however, the participants were all in the chronic phase where it is known that there is little recovery if there is no intervention. The ClinicHEAD thus served as a training and getting to know the system phase, all participants had the same training and so had the same base for the HomeHEAD phase that instead was experimental and had the purpose of inquiring upon the effect of bringing a known system home for further training.

### HomeHEAD, Usual Care, and Maintenance at Follow Up (T2-T1, T3-T1)

The main results of our study are that HomeHEAD impacted on longterm maintenance of functional mobility. Six months after ClinicHEAD, at 3 months follow up of HomeHEAD, the HH_group had increased the distance they could walk in 2 min while the UC_group had lost some mobility, resulting in a significant difference between the two groups in preserving mobility results over the 6 months. This is an important result since mobility and gait speed are important aspects of health and have been demonstrated to be predictive of life participation and need for hospital recovery (44, 45). Thus, potentially, bringing the HEAD system home preserved mobility functions in our HH_group and delayed the need for further care. Moreover, there was an improvement in memory at 6 months from ClinicHEAD only in the HH_group, indicating further longterm benefit on memory from bringing the HEAD system home.

Regarding the effect of bringing the HEAD system home for daily weekday training, there was no further increase in any motor or cognitive outcome immediately after 3 months of HomeHEAD. Importantly, there were no differences in maintenance of ClinicHEAD benefits between the participants doing HomeHEAD training and those that followed usual care. Both groups preserved the results achieved. One of the reasons for no further improvements in the HH group may be due to effects of any intervention being largest in the first couple of weeks of intervention, such as, that seen by Krakauer et al. (46). It may be an unrealistic goal to expect further significant improvement in motor and cognitive outcomes after 4 weeks of ClinicHEAD treatment.

In their review Aminov et al. (41) looked at follow up data from VR training and interestingly found no difference between effects immediately following training and follow up end point. The studies reviewed all had between 6 and 12 weeks follow up so our finding of preserving of initial VR training results at 3 months for the UC_group are in line with the review's findings. At 6 months without training there was, however, a detoriation of mobility in the UC_group compared to the HH_group that was only at 3 months follow up from end of HomeHEAD. Regarding the longer-term efficiency of the HomeHEAD approach, this would have to be studied in future studies with longer than 3 months follow up.

### General Discussion and Limitations

While most people with chronic neurological disorders experiment the major part of functional recovery while in clinic, many could continue to improve, or at least, preserve abilities, over longer time periods. Patient engagement in the paradigm of rehabilitation in continuity of care is an important issue. With motivated engagement of the chronic patient they can become main actors responsible for their life and health care and can be accompanied by the health care system rather than being dependent on it (22). Telerehabilitation using VR systems is useful for manipulating and augmenting the interaction between the user and the environment, with the objective of impacting on neuromotor and cognitive recovery, ultimately leading to increased activity and daily life participation. The use of VR systems in rehabilitation is consequently an important option both during recovery and in continuity of care. In particular, they become essential for persons living in rural zones or persons that for some reason have limited access to rehabilitation care.

Our VR HEAD study is a multicenter study that was carried out by a multidisciplinary team. The training protocol was developed in collaboration between different health professionals and was aimed at improving many aspects of health of the individual, motor and non-motor, resulting in a complex intervention in line with that recommended by Langhorne^.^(44). The HEAD training strategies allowed an interplay between therapeutic goals and individual abilities, with regular adaptation of difficulty so that it progressively demanded more of the person in t**r**aining (47). At the end of the follow up period there was an important maintenance of mobility in the participants that brought the system home, an aspect of functioning that is partly indicative of independence in daily activities and mobility out of the home. This indicates the HEAD multidisciplinary approach to rehabilitation may be well-suited for continuity of care, it is ecologically valid and effective without interfering with the persons everyday living activities and can potentially progressively augment autonomy and ability of the person in training (22, 23, 48).

Importantly, the HEAD approach was viewed positively by most of the persons playing it with no distinction of age or sex and the majority of the persons using it during the in-clinic phase were convinced they would use it in the home setting if the opportunity arose. One of the major benefits of the HEAD VR system is in fact the possibility to continue rehabilitation under occasional supervision of health professionals (theoretically infinitely) for much longer than would otherwise be possible. With the VR serious games approach therapy can be incorporated into daily home and work activities thus reducing cost to society in terms of home assistance or absence from work. Further, the opportunity of longer rehabilitation time will allow bigger improvement and/or longer maintenance of function which is important for persons with neurological disorders that need to both consolidate the results obtained during recovery in rehabilitation centers and continue improving their function in their daily habitat.

A strength of the study approach used in the present study is the fact that both groups carried out the same VR rehabilitation during the 4 weeks in clinic, while only after that they are split up into an experimental and a control group, thus the groups share a phase in rehabilitation period. Any change or better maintaining of these in clinic treatment effects can thus be attributed to the HomeHEAD intervention rather than being an effect of beginning a new rehabilitation activity as described by Dobkin and Carmichael (49).

Our pilot study, however, has several limitations, first of all for not being a random controlled trial with equal sample sizes. Generalization of results is also limited to persons with chronic stroke that adhere to the protocol and are able to stand at least 30 s. Another limit, shared with most other studies in the literature studying the effect of VR home rehabilitation, is that the sample participating was small so conclusions can only be indicative as to efficiency of the approach. However, the study has provided indications of feasibility and an estimation of the potential efficiency and effect sizes of a motor and non-motor telerehabilitation protocol (HEAD) for people with chronic stroke that will be useful in future larger Phase III trials.

Yet another limitation might be the use of a passive control group in the HomeHEAD phase, however, Aminov et al. in their systematic review (41), found no difference in effect sizes of virtual reality outcomes when compared to either active and passive control groups suggesting that the use of a passive control group may not have impacted on the outcome.

### Conclusion

There was an increase in most evaluated motor and cognitive outcomes following 12 in-Clinic sessions of the HEAD VR approach indicating that with this global approach to rehabilitation it is possible to impact on many of the deficits that persons with chronic stroke have to live with. Importantly, use of the approach in continuity of care may have increased longterm maintenance of mobility, an important aspect of daily functioning. Over the lifetime of having a neurological disorder multidisciplinary rehabilitation is an integral part of improvement and maintaining of functionality and likely, virtual reality approaches will be part of future longterm neurorehabilitation solutions.

## Data Availability Statement

The raw data supporting the conclusions of this article will be made available by the authors, without undue reservation.

## Ethics Statement

The studies involving human participants were reviewed and approved by IRCCS Don Gnocchi Foundation Ethical Committee, Intercompany of Lecco, Como, and Sondrio Ethical Committee, Intercompany of Citta della Salute e della Scienza of Turin Ethical Committee. The patients/participants provided their written informed consent to participate in this study.

## Author Contributions

FB, MS, and FM conceived the study. CP, CC, CG, and TB recruited sample and did clinical evaluation. JJ, PG, GPa, and GPe collected data. JJ, SD, SI, and FB performed analysis and interpreted the results. JJ, SI, and FB wrote the first draft of the manuscript. All authors reviewed and approved the final manuscript.

## Head Study Group

Aggujaro S., Barra G., Bellomo M., Bertoni R., Boccini S., Bonanima M., Borgogno P., Canobbio S., Castagna A., Castiglioni C., Covarrubias M., Del Principe A., Enei L., Ferrari A., Ferrarin M., Fini M., Gencarelli N., Giordano A., Manfredini C., Marino C., Martina L., Mendozzi L., Mocarelli P., Montesano A., Nemni R., Peverelli M., Proserpio D., Pugnetti L., Ripamonti E., Rossini M., Rossini M., Ruffin G., Saibene F. L., Trombini D., Zanfini A.

## Conflict of Interest

The authors declare that the research was conducted in the absence of any commercial or financial relationships that could be construed as a potential conflict of interest.
